# Assessment of Public Knowledge and Awareness on First-Aid Management of Epistaxis in Makkah, Saudi Arabia

**DOI:** 10.7759/cureus.47945

**Published:** 2023-10-30

**Authors:** Raghad Y Shosho, Raghad O Al-Masoudi, Abdulwahab I Kamal, Abdulrahman F Kabli, Ahmed J Alzahrani, Hawra A Almusallam, Raad A Alqurashi, Abdulaziz T Baghdadi, Salman A Alosaimi, Mokhtar Shatla

**Affiliations:** 1 Medicine and Surgery, Umm Al-Qura University, Makkah, SAU; 2 College of Medicine and Surgery, Arabian Gulf University, Manam, BHR; 3 Otolaryngology, Head and Neck Surgery, King Abdullah Medical City, Makkah, SAU; 4 College of Medicine and Surgery, Dar Al Uloom University, Riyadh, SAU; 5 Emergency Medicine, AlNoor Specialist Hospital, Makkah, SAU; 6 College of Medicine and Surgery, Umm Al-Qura University, Makkah, SAU; 7 Family Medicine, Community Medicine, and Pilgrims Healthcare, College of Medicine, Umm Al-Qura University, Makkah, SAU

**Keywords:** kingdom of saudi arabia (ksa), public, first-aid, makkah region, epistaxis

## Abstract

Background: Epistaxis is a common medical problem requiring first aid. However, public awareness of proper first-aid techniques for epistaxis is limited. This study assessed the knowledge and attitudes toward first-aid epistaxis management among the general population in the Makkah region.

Methods: A cross-sectional online survey was conducted with 1,259 participants from various regions in Makkah. The survey assessed participants’ epidemiological information, experience with epistaxis, and knowledge of proper first-aid techniques. The knowledge scores were calculated and categorized as either good or poor. Associations between the variables and predictors of good knowledge were analyzed.

Result: Overall knowledge of proper epistaxis first aid was poor, with only 467 (37.1%) demonstrating good knowledge. Women, healthcare workers, and those with prior epistaxis showed higher odds of having good knowledge than their counterparts. Relatives/friends and social media were cited as the main knowledge sources, rather than healthcare providers.

Conclusion: Public awareness of appropriate first-aid epistaxis techniques in Makkah is inadequate. Targeted educational interventions involving healthcare providers are needed to improve the first-aid management of this common medical issue.

## Introduction

Epistaxis, commonly known as a nosebleed, is a frequent medical concern encountered in emergency departments (ED) owing to its abrupt onset and potential severity. Various factors can precipitate epistaxis, including local, systemic, or environmental triggers, such as trauma, infections, allergies, and elevated blood pressure [1]. The nose is richly vascularized and primarily supplied by the internal and external carotid arteries, respectively. The primary cause of epistaxis is the rupture of blood vessels within the nasal mucosa, with Keisselbach’s plexus being the most anatomically prevalent site of occurrence [1-3].

Statistics indicate that approximately 60% of the general population experiences epistaxis, with 10% of the cases being severe enough to warrant medical intervention. Epistaxis predominantly affects children and older adults [2,4,5]. While most cases are benign and can be managed at home, certain cases necessitate medical attention [6]. However, the general population needs adequate knowledge of managing acute cases that do not require medical care [4]. Additionally, all ED physicians should be proficient in first-aid measures for epistaxis [5].

Proper first-aid techniques involve sitting the patient upright and tilting the head forward to protect the airway. Applying pressure to Kiesselbach’s plexus by pinching the nasal ala against the septum for 10-15 minutes addressed 95% of the bleeding episodes. These procedures often halt active nosebleeds when correctly executed [5]. The misconception of tilting the head backward and applying pressure on the nasal bones may also be common among the public [5]. Notably, a prospective cross-sectional study conducted among medical practitioners in Kenya revealed a deficiency in knowledge and attitudes toward the first-aid management of epistaxis [7]. Prior studies in Saudi Arabia have advocated for public education on epistaxis first-aid management through camps and workshops [4,8].

In most instances, epistaxis does not result in complications; however, when it does occur, it can be severe. Complications associated with epistaxis include septal hematoma, external nasal deformity, balloon migration, hemorrhagic shock, and mucosal pressure necrosis. Injuries can be significantly reduced if first aid is provided immediately, reducing the disease course and complications [2].

A study on the older adult population emphasized the need for early intervention and timely administration of first-aid measures to prevent complications [9]. Furthermore, literature reviews underscore the necessity of standardized guidelines and training first-aid providers in managing epistaxis emergencies [2,9].

Although some studies have investigated the awareness and first-aid management of epistaxis in Saudi Arabia, there is a lack of such research in the Makkah region. Therefore, the primary objective of this study was to assess the knowledge and attitude toward first-aid management of epistaxis among the general population in the Makkah region.

## Materials and methods

This study used a cross-sectional design to examine the participants’ knowledge of first-aid management for epistaxis in the Makkah region. A questionnaire was developed (refer to appendix), and 400 participants were recruited for an adequate sample size. However, over the course of the research period, 1,259 participants were included in the study.

We invited participants through an online survey distributed across various social media platforms such as WhatsApp, Telegram, Twitter, and Facebook. This method allowed for a diverse sample of individuals from different geographical areas, age groups, and cultural backgrounds.

The questionnaire used in the survey was carefully constructed and included sociodemographic information, such as sex, age, occupation, and residence. In addition to gathering information on participants’ knowledge of epistaxis (e.g., causes, symptoms, and risk factors), the survey also explored their attitudes and practices regarding first-aid management of epistaxis. The myths were assessed using a questionnaire.

Measures were taken to reduce bias and maintain ethical standards to ensure the validity and reliability of the collected data. The survey was developed based on existing literature to ensure it encompassed relevant and up-to-date information [1]. Moreover, to prevent duplicate responses and ensure accuracy, mechanisms were implemented to prevent individuals from completing the surveys multiple times.

Emphasis was placed on adherence to ethical guidelines throughout the study. Informed consent was obtained from all participants, and confidentiality was maintained. The collected data were anonymized and stored securely to protect the participants’ confidentiality and privacy. This study was approved by the Biomedical Ethics Committee of Umm Al-Qura University.

By employing a rigorous methodology and maintaining strict ethical standards, this study aimed to provide in-depth and accurate insights into the participants’ knowledge of epistaxis first aid.

^Scoring^

We calculated the knowledge score based on seven items in the current study. Each response was coded as one for correct answers and zero for incorrect answers. The overall knowledge score was calculated by summing the relevant correct responses. Therefore, the scores ranged from zero to seven, with higher scores indicating higher knowledge. A percentage score was calculated, and the participants’ knowledge was categorized as poor (percentage score: ≤50%) or good (percentage score: >50%)

^Statistical analysis^

Statistical analyses were performed using RStudio software (R version 4.3.0) (RStudio, PBC, Boston, MA). Categorical variables are presented as frequencies and percentages. We performed normality testing on the overall knowledge score, which revealed a non-normally distributed variable (Shapiro-(A3) Wilk test, p<0.001). Therefore, we presented the knowledge score as medians and interquartile ranges (IQR). Factors associated with epistaxis and knowledge levels (poor or good) were assessed using Pearson’s chi-square or Fisher’s exact tests, whenever applicable. Predictors of good knowledge were assessed by constructing a multivariable logistic regression model using knowledge level (poor vs. good) as the dependent variable and the significantly associated variables from the inferential analysis as independent variables. The results are presented as odds ratios (ORs) and 95% confidence intervals (CIs). Statistical significance was set at p<0.05.

## Results

We received 1,322 responses on the online platform, and 63 responses with missing data were excluded from the analysis. Therefore, we analyzed data from 1,259 participants, of which the majority were female (838, 66.6%) and within the age range of 18-35 years were 820 (65.1%). A significant proportion of the respondents resided in Makkah (656, 52.1%). Regarding marital status, a substantial proportion identified as single (696, 55.3%). Regarding educational level, a significant majority had a postgraduate education (74.9%), and most participants reported being unemployed (607, 48.2%). Furthermore, the majority of respondents had a monthly income below 5,000 SAR (666, 52.9%) (Table 1).

**Table 1:** Sociodemographic characteristics of the respondents

Characteristic	N (%)
Sex	
Male	421 (33.4)
Female	838 (66.6)
Age (years)	
18-35	820 (65.1)
36-50	302 (24.0)
>50	137 (10.9)
Place of residence	
Taif	251 (19.9)
Jeddah	264 (21.0)
Makkah	656 (52.1)
Rural regions	88 (7.0)
Marital status	
Single	696 (55.3)
Married	462 (36.7)
Widow	25 (2.0)
Divorced	76 (6.0)
Educational level	
Primary school	9 (0.7)
Intermediate school	45 (3.6)
High school	262 (20.8)
Postgraduate	943 (74.9)
Employment status	
Unemployed	607 (48.2)
Healthcare worker	136 (10.8)
Non-healthcare worker	516 (41.0)
Monthly income (SAR)	
<5,000	666 (52.9)
5,000-15,000	418 (33.2)
>15,000	175 (13.9)

**Figure 1:**
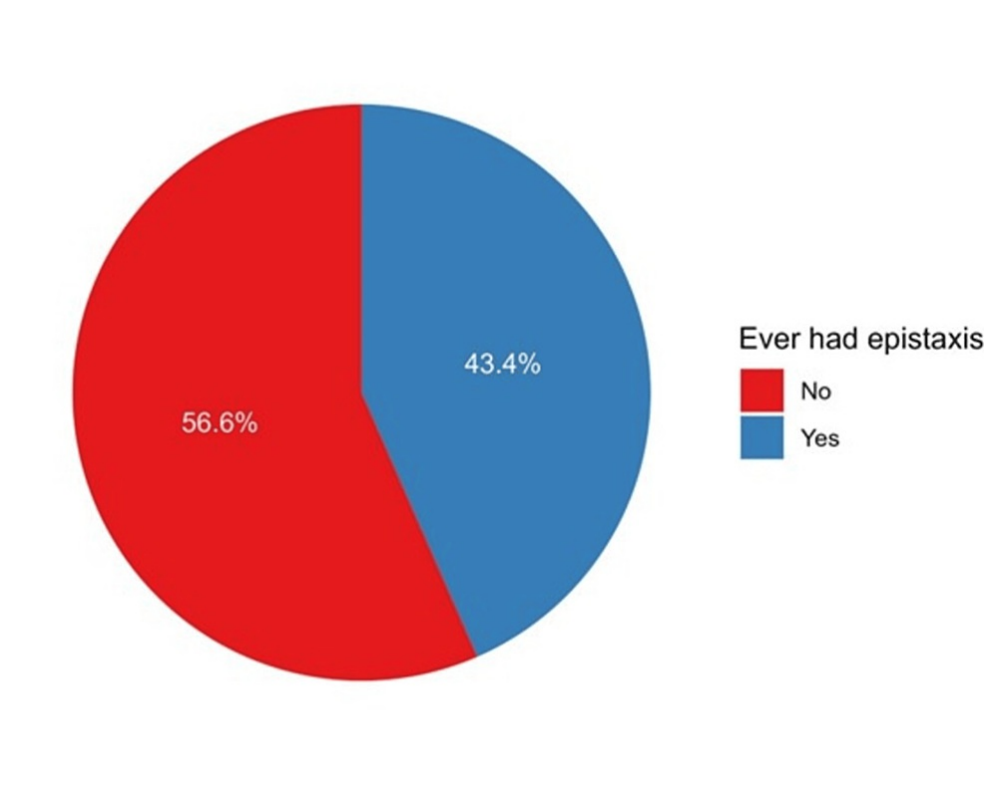
The proportions of participants who experienced epistaxis

A total of 547 respondents had experienced epistaxis (43.4%) (Figure 1).

There were significant differences in the proportion of participants with epistaxis in terms of marital status (p=0.016), employment status (p<0.001), and monthly income (p<0.001; Table 2).

**Table 2:** Factors associated with having epistaxis

Characteristic	Ever had epistaxis		p-value
	No, N=712, N (%)	Yes, N=547, N (%)	
Gender			0.710
Male	235 (55.8)	186 (44.)	
Female	477 (56.9)	361 (43.1)	
Age (years)			0.342
18 to 35	476 (58.0)	344 (42.0)	
36 to 50	162 (53.6)	140 (46.4)	
>50	74 (54.0)	63 (46.0)	
Place of residence			0.796
Taif	135 (53.8)	116 (46.2)	
Jeddah	152 (57.6)	112 (42.4)	
Makkah	374 (57.0)	282 (43.0)	
Rural regions	51 (58.0)	37 (42.0)	
Marital status			0.016
Single	396 (56.9)	300 (43.1)	
Married	270 (58.4)	192 (41.6)	
Widow	16 (64.0)	9 (36.0)	
Divorced	30 (39.5)	46 (60.5)	
Educational level			0.117
Primary school	4 (44.4)	5 (55.6)	
Intermediate school	18 (40.0)	27 (60.0)	
High school	151 (57.6)	111 (42.4)	
Postgraduate	539 (57.2)	404 (42.8)	
Employment status			<0.001
Unemployed	376 (61.9)	231 (38.1)	
Healthcare worker	70 (51.5)	66 (48.5)	
Non-healthcare worker	266 (51.6)	250 (48.4)	
Monthly income (SAR)			<0.001
<5,000	407 (61.1)	259 (38.9)	
5,000 to 15,000	223 (53.3)	195 (46.7)	
>15,000	82 (46.9)	93 (53.1)	

Table 3 shows the proportion of correct answers among the participants for various knowledge items related to epistaxis first-aid management. A significant majority of the respondents recognized the importance of first-aid measures for epistaxis (992, 78.8%), whereas 585 (46.5%) correctly identified tilting the head forward as the best position to stop epistaxis. Less than half of the participants correctly acknowledged that applying pressure on the nose could stop epistaxis (699, 55.5%) and identified the lower part (cartilage) of the nose as the appropriate location for applying pressure (392, 31.1%). Only 10.4% of the respondents correctly knew that pressure should be applied for 11-15 minutes. However, regarding the right time to seek medical attention, a significant number of participants (624, 49.6%) correctly indicated that it should be performed after >20 minutes of unsuccessful bleeding control. Finally, almost one-fifth of the respondents recognized that nasal drops could help stop epistaxis (247, 19.6%) (Table 3).

**Table 3:** Participants’ responses to knowledge items *the correct answers

Characteristic	N (%)
First-aid measures are important for epistaxis	
No	147 (11.7)
Yes*	992 (78.8)
Do not know	120 (9.5)
The best position to stop epistaxis	
Lie down with your feet up	88 (7.0)
Tilt the head forward*	585 (46.5)
Tilt the head back	349 (27.7)
I do not know	237 (18.8)
Applying pressure on the nose can stop epistaxis	
No	303 (24.1)
Yes*	699 (55.5)
Do not know	257 (20.4)
Part of the nose that you will apply pressure on it	
The lower part (cartilage)*	392 (31.1)
Upper part (bone)	469 (37.3)
I do not know	398 (31.6)
How long you will apply pressure on your nose?	
5 minutes	682 (54.2)
6-10 minutes	405 (32.2)
11-15 minutes*	131 (10.4)
16-20 minute	41 (3.3)
What will be the right time to go to?	
More than 20 minutes*	624 (49.6)
More than 40 minutes	206 (16.4)
More than 60 minutes	151 (12.0)
I will not go to the ER at all	235 (18.7)
Do not know	43 (3.4)
Nasal drops can help stop epistaxis	
No	456 (36.2)
Yes*	247 (19.6)
Do not know	556 (44.2)

**Figure 2:**
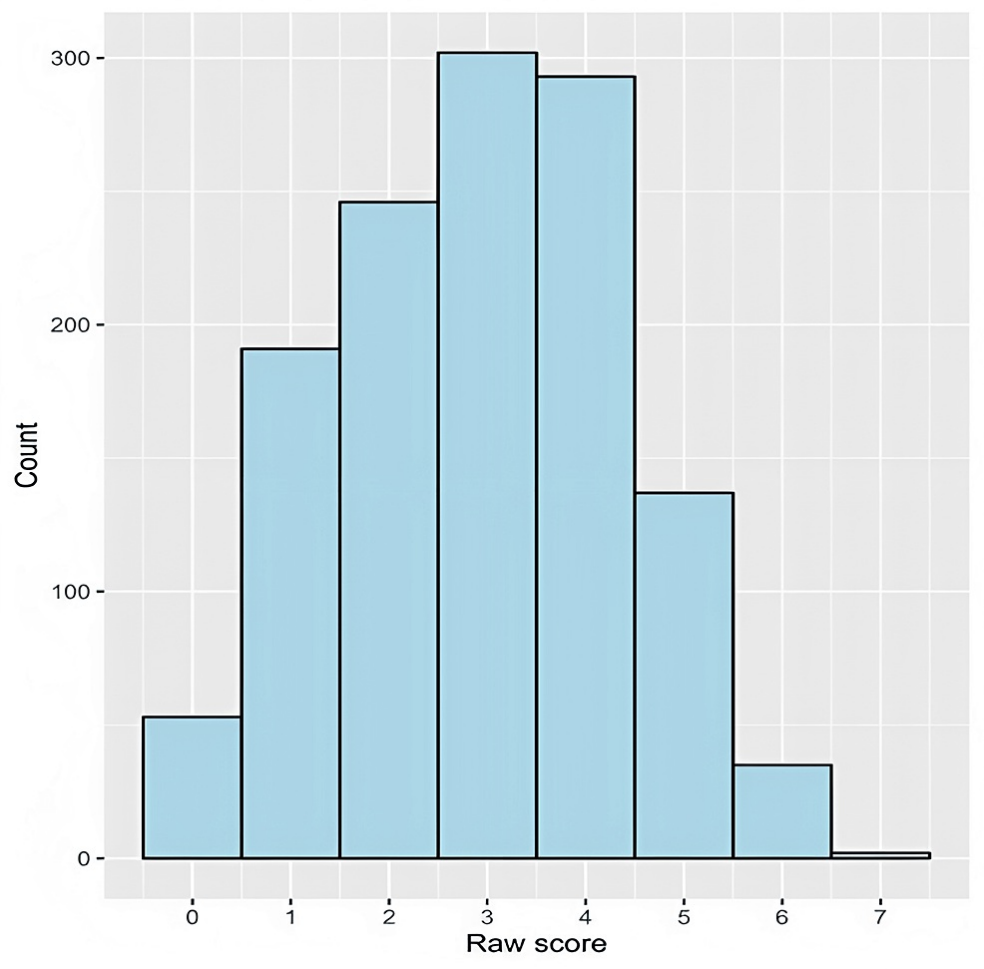
A histogram showing the frequency distribution of the raw knowledge score

The median (IQR) knowledge score for all participants was 3.0 (2.0-4.0). The frequency distribution of knowledge scores is shown in Figure 2.

A total of 467 respondents had scores >50%, indicating good knowledge (representing 37.1% of the sample). The results of the inferential analysis revealed that sex exhibited a significant association with knowledge level (p<0.001), as a higher proportion of females (341, 40.7%) possessed good knowledge than males (126, 29.9%). Employment status also displayed a significant association with knowledge level (p<0.001), with healthcare workers demonstrating a higher proportion of good knowledge (80, 58.8%) than non-healthcare workers (166, 32.2%) and unemployed respondents (221, 36.4%). Moreover, a history of epistaxis was significantly associated with the knowledge level (p<0.001), as individuals who had previously experienced epistaxis exhibited a greater proportion of good knowledge (257, 47.0%) than those who had not (210, 29.5%) (Table 4).

**Table 4:** Factors associated with good knowledge regarding epistaxis Predictors for possessing good knowledge regarding epistaxis

Characteristic	Knowledge level		p-value
	Poor, N=792, N (%)	Good, N=467, N (%)	
Sex			<0.001
Male	295 (70.1)	126 (29.9)	
Female	497 (59.3)	341 (40.7)	
Age (years)			0.091
18-35	502 (61.2)	318 (38.8)	
36-50	193 (63.9)	109 (36.1)	
>50	97 (70.8)	40 (29.2)	
Place of residence			0.305
Taif	150 (59.8)	101 (40.2)	
Jeddah	158 (59.8)	106 (40.2)	
Makkah	426 (64.9)	230 (35.1)	
Rural regions	58 (65.9)	30 (34.1)	
Marital status			0.627
Single	433 (62.2)	263 (37.8)	
Married	298 (64.5)	164 (35.5)	
Widow	17 (68.0)	8 (32.0)	
Divorced	44 (57.9)	32 (42.1)	
Educational level			0.090
Primary school	7 (77.8)	2 (22.2)	
Intermediate school	27 (60.0)	18 (40.0)	
High school	181 (69.1)	81 (30.9)	
Postgraduate	577 (61.2)	366 (38.8)	
Employment status			<0.001
Unemployed	386 (63.6)	221 (36.4)	
Healthcare worker	56 (41.2)	80 (58.8)	
Non-healthcare worker	350 (67.8)	166 (32.2)	
Monthly income (SAR)			0.773
<5,000	425 (63.8)	241 (36.2)	
5,000-15,000	258 (61.7)	160 (38.3)	
>15,000	109 (62.3)	66 (37.7)	
Ever had epistaxis			<0.001
No	502 (70.5)	210 (29.5)	
Yes	290 (53.0)	257 (47.0)	

Table 5 presents the predictors of good knowledge regarding first-aid management of epistaxis among the study participants. Females demonstrated higher odds of possessing good knowledge compared to males (OR=1.66, 95% CI: 1.28-2.15, p<0.001). Moreover, employment status played a significant role in predicting knowledge levels, with healthcare workers having substantially greater odds of good knowledge (OR=2.43, 95% CI: 1.65-3.60, p<0.001), while non-healthcare workers exhibited lower odds (OR=0.77, 95% CI: 0.60-0.99, p=0.044). Furthermore, individuals who had experienced epistaxis in the past displayed higher odds of good knowledge (OR=2.20, 95% CI: 1.73-2.79, p<0.001; Table 5).

**Table 5:** Predictors of good knowledge regarding epistaxis OR, odds ratio; CI, confidence interval

Characteristic	OR	95% CI	p-value
Gender			
Male	Reference	Reference	
Female	1.66	1.28, 2.15	<0.001
Employment status			
Unemployed	Reference	Reference	
Healthcare worker	2.43	1.65, 3.60	<0.001
Non-healthcare worker	0.77	0.60, 0.99	0.044
Ever had epistaxis			
No	Reference	Reference	
Yes	2.20	1.73, 2.79	<0.001

Sources of knowledge

**Figure 3:**
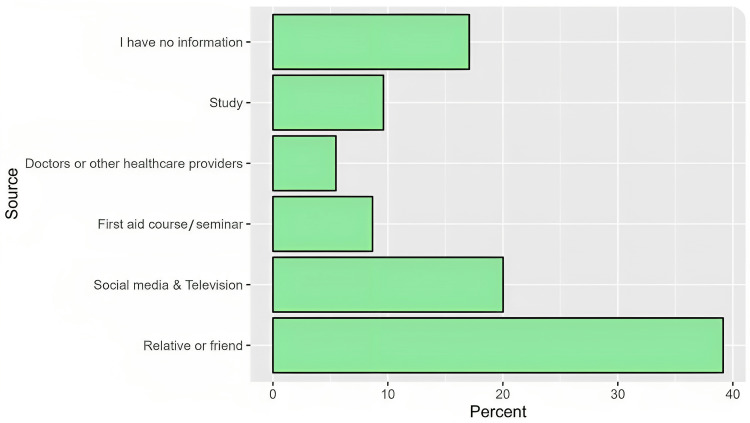
Sources of information regarding epistaxis

Based on the participants’ responses, the most commonly reported sources of knowledge regarding epistaxis first-aid management were relatives or friends (39.2%), social media, and television (20.0%), whereas 5.5% of the respondents only appreciated the role of doctors or healthcare providers (Figure 3).

## Discussion

This study aimed to determine the extent to which the general public knew about epistaxis, an acute illness characterized by nasal bleeding. The sight of blood gushing out of the nose can be horrifying and cause anxiety, even though most epistaxis events are benign and treatable with first aid. Therefore, educating the public on the fundamental treatment of epistaxis is crucial. In this study, most participants acknowledged the significance of epistaxis first-aid measures (78.8%), while 46.5% correctly identified tilting the head forward as the optimal position to end epistaxis. Less than half of the participants (55.5%) accepted that applying pressure to the nose ceased epistaxis, and only 31.1% correctly identified the cartilage at the bottom of the nose as the proper place to apply pressure. Only 10.4% of the respondents correctly identified the recommended pressure application time of 11-15 minutes.

There was a substantial correlation between employment status and knowledge level, with healthcare workers having a larger percentage of good knowledge (58.8%) than non-healthcare workers (32.2%) and unemployed respondents (36.4%). Alyahya et al. (2019) found that only 45% of medical students who participants knew where to apply pressure correctly and that 55.5% knew about bending the bleeding patient forward [10]. The results presented herein have a wide range of applications. One study has revealed that approximately 80% of participants know the correct procedure [4]. However, as these investigations were conducted with medical students, they were expected to possess more knowledge. In another study by Saleem (2018), only 6% of the patients knew the optimal location and method for applying nasal pressure [11]. Strachan and England (1998) reported 36% method awareness, which was comparable but marginally better [9]. Almulhim et al. reported that 64.5% of the population believed that compressing the nose was beneficial in stopping bleeding, and 58.1% stated that leaning forward was the most effective position to stop bleeding [12].

In this study, the prevalence of self-reported epistaxis was 43.4%, which was lower than that reported in another study (71%) [13]. Additionally, the prevalence of epistaxis in this study was significantly higher in females than in males, in contrast to the findings of another study conducted in Saudi Arabia [14].

Employment status significantly influenced the likelihood of having strong knowledge, with healthcare professionals having significantly higher odds than non-healthcare workers [15]. Additionally, those who had previously encountered epistaxis were more likely to have a sound understanding. According to Al-Shehri et al. (2021), there were various responses from respondents regarding first-aid management of epistaxis [1]. They knew what epistaxis was and how to treat it with first aid but were unsure about the proper application techniques. For information on at-home epistaxis management, the majority turn to the internet and social media. In 2022, Al-Dhafiri et al. reported that epistaxis was one of the most frequently reported emergencies in the ear, nose, and department [5]. The causes of epistaxis ranged from minor, such as picking one’s nose or breathing dry air, to serious, such as infections or high blood pressure, to potentially fatal, such as cancers. Epistaxis occurs in the general population at a rate of 60%, of which 10% requires medical attention and is somehow tied to the duties of family care and family physicals.

According to Mohammad et al. (2020), 80-85% of customers are familiar with ailments such as epistaxis and are aware that it may be a chronic illness [4]. Most otorhinolaryngology patients experience one or more episodes of epistaxis during their lifespan. Epistaxis is the name used for bleeding from the nose, nasal cavity, or nasopharynx due to a rupture of the blood artery inside the nose. Although epistaxis is typically mild and curable, it can occasionally become severe and even life-threatening.

First-aid management and other emergency preventive tactic-related information are necessary to increase responsibility and raise one’s social position. Regardless of age, treatment for epistaxis starts with stabilization, diagnosis of the cause of the hemorrhage, and treatment of the underlying cause. To reduce mortality and morbidity, it is crucial that the public is aware of and knowledgeable about basic first-aid methods for managing hemorrhage episodes because the majority of epistaxis incidents occur in a medical context.

One of the strengths of this study is its identification of unexplored knowledge and attitudes toward epistaxis in the Makkah region, as well as its large sample size of 1,259 participants compared with other studies in Saudi Arabia. Data collection was facilitated by disseminating online surveys across various social media platforms, thereby enhancing the generalizability of the findings. The residents of Makkah stand to gain diverse benefits from the insights gained through this study.

Nevertheless, our study had some limitations. The reliance on self-reporting and potential electronic interference with the questionnaire on social media platforms may have led to underreporting by participants. Additionally, this investigation concentrated exclusively on the Makkah region, necessitating caution when generalizing the results to the entire Saudi Arabian population for a comprehensive understanding of epistaxis. Furthermore, this study employed a cross-sectional design, suggesting the need for cohort studies to provide deeper insights into this issue.

Considering our findings, we advocate the expanded role of healthcare professionals in Makkah, particularly those whose knowledge is not primarily derived from medical training. Additionally, we recommend implementing comprehensive social media awareness programs tailored to the specific demographics of the target population. It is imperative to develop awareness campaigns in venues such as malls, schools, and hospitals, with a focus on males, who exhibit discernible knowledge gaps compared with their female counterparts.

## Conclusions

This study aimed to assess the knowledge and attitudes of the general population in the Makkah region regarding first-aid management of epistaxis. The findings revealed gaps in knowledge among the participants, particularly regarding proper positioning, duration of pressure application, and appropriate location for applying pressure. However, a considerable proportion of participants recognized the importance of first-aid measures for epistaxis and the right time to seek medical attention. Factors such as sex, employment status, and previous experience with epistaxis were associated with higher levels of knowledge. The most common sources of knowledge were relatives, friends, and social media rather than healthcare providers.

## Appendices

The questionnaire was translated into the Arabic language to collect data from the population 

Part 1: Demographic data

Q1 Are you living in the Makkah region?

o        Yes

o        No

Q2 Gender?

o        female

o        Male

Q3 Age?

o        <18

o        19-29

o        30-39

o        40-49

o        50 or above

Q4 Living in?

o        City

o        Village

Q5 Marital status?

o        Single

o        Married

o        Divorced

o        Widow

Q6 Educational level?

o        Primary school

o        Intermediate school

o        High school

o        Postgraduate

Q7 Career?

o        Healthcare worker

o        Non-healthcare worker

Q8 Monthly income?

o        <5000 SR

o        5000-15000 SR

o        >15000 SR

Part 2: Knowledge, attitude, and practice of first-aid management of epistaxis

Q1 Do you suffer from epistaxis?

o   Yes

o   No

Q2 Do you think first-aid measures are important for epistaxis?

o        Yes

o        No

o        Do not Know

Q3 What is the best position to stop epistaxis?

o        Lie down with your feet up

o        Tilt the head forward

o        Tilt the head back

o        Do not know

Q4 Applying pressure on the nose can stop epistaxis?

o        Yes

o        No

o   Do not know

Q5 Which part of the nose will you apply pressure during epistaxis?

o        The lower part (cartilage)

o        Upper part (bone)

o        Do not know

Q6 How long will you apply pressure on your nose?

o        5 minutes

o        6-10 minutes

o        11-15 minutes

o        16-20 minutes

Q7 What will be the right time to go to the ER?

o        Once the epistaxis happen

o        If the bleeding does not stop after more than 20 minutes

o        If the bleeding does not stop after more than 40 minutes

o        If the bleeding does not stop after more than 60 minutes

o        I will not go to the ER at all

o        Do not know

Q8 Source of information?

o        Relative or friend

o        Social media and television

o        First-aid course seminar

o        Doctors and other healthcare workers

o        I do not have the information

o        Study
